# Cholelithiasis and cholecystectomy increase the risk of gastroesophageal reflux disease and Barrett’s esophagus

**DOI:** 10.3389/fmed.2024.1420462

**Published:** 2024-07-18

**Authors:** Yu Huang, Yicong Cai, Yingji Chen, Qianjun Zhu, Wei Feng, Longyu Jin, Yuchao Ma

**Affiliations:** ^1^Department of Cardiothoracic Surgery, Third Xiangya Hospital of Central South University, Changsha, China; ^2^Department of Gastrointestinal Surgery, Third Xiangya Hospital of Central South University, Changsha, China

**Keywords:** cholelithiasis, cholecystectomy, gastroesophageal reflux disease, Barrett’s esophagus, esophageal adenocarcinoma, meta-analysis, Mendelian randomization

## Abstract

**Background:**

Cholelithiasis or cholecystectomy may contribute to the development of gastroesophageal reflux disease (GERD), Barrett’s esophagus (BE), and esophageal adenocarcinoma (EAC) through bile reflux; however, current observational studies yield inconsistent findings. We utilized a novel approach combining meta-analysis and Mendelian randomization (MR) analysis, to assess the association between them.

**Methods:**

The literature search was done using PubMed, Web of Science, and Embase databases, up to 3 November 2023. A meta-analysis of observational studies assessing the correlations between cholelithiasis or cholecystectomy, and the risk factors for GERD, BE, and EACwas conducted. In addition, the MR analysis was employed to assess the causative impact of genetic pre-disposition for cholelithiasis or cholecystectomy on these esophageal diseases.

**Results:**

The results of the meta-analysis indicated that cholelithiasis was significantly linked to an elevated risk in the incidence of BE (RR, 1.77; 95% CI, 1.37–2.29; *p* < 0.001) and cholecystectomy was a risk factor for GERD (RR, 1.37; 95%CI, 1.09–1.72; *p* = 0.008). We observed significant genetic associations between cholelithiasis and both GERD (OR, 1.06; 95% CI, 1.02–1.10; *p* < 0.001) and BE (OR, 1.21; 95% CI, 1.11–1.32; *p* < 0.001), and a correlation between cholecystectomy and both GERD (OR, 1.04; 95% CI, 1.02–1.06; *p* < 0.001) and BE (OR, 1.13; 95% CI, 1.06–1.19; *p* < 0.001). After adjusting for common risk factors, such as smoking, alcohol consumption, and BMI in multivariate analysis, the risk of GERD and BE still persisted.

**Conclusion:**

Our study revealed that both cholelithiasis and cholecystectomy elevate the risk of GERD and BE. However, there is no observed increase in the risk of EAC, despite GERD and BE being the primary pathophysiological pathways leading to EAC. Therefore, patients with cholelithiasis and cholecystectomy should be vigilant regarding esophageal symptoms; however, invasive EAC cytology may not be necessary.

## Introduction

Esophageal adenocarcinoma is the seventh most common cancer globally and the sixth leading cause of mortality due to cancer, making it an important topic for research and study (1). Despite advances in early detection and treatment, the five-year survival in EAC remains less than 20% (2). Reflux is a major risk factor for the EAC (3). A prolonged or severe gastroesophageal reflux disease (GERD) increases the risk of EAC by up to 40 times (4). Chronic GERD causes the metaplasia of esophageal squamous cell mucosa to specialized columnar epithelium, called Barrett’s esophagus (BE), and it is the main pathophysiological route of EAC (5). The three esophageal diseases, GERD, BE, and EAC, are closely related. GERD is a major cause of BE, which in turn is a major risk factor for EAC (6). Bile reflux is the most common feature of GERD, other than acid reflux (7). The current consensus suggests that acid and bile reflux collaborate to induce esophagitis and induce metaplasia of the EAC sequence toward atypical hyperplasia (8). In addition, studies have shown that the concentration of bile in the esophagus of patients after cholecystectomy is significantly increased (9).

Cholelithiasis is one of the most common diseases of the digestive system (10). Cholecystectomy is the standard treatment for symptomatic cholelithiasis. However, the impact of cholecystectomy on quality of life remains a critical clinical question in most patients (11), considering the relationship between cholelithiasis or cholecystectomy and bile reflux (12, 13). It can be assumed that both cholelithiasis and cholecystectomy can affect the occurrence of GERD, BE, and EAC by promoting bile reflux. However, clinical studies on the relationship between cholelithiasis, cholecystectomy, and esophageal reflux disease have provided conflicting results. Two studies reported an increased incidence of BE in patients with cholelithiasis (14, 15). Several studies have shown that cholecystectomy increases the risk of GERD and EAC (12, 16–19). However, in many studies, there was no association between the risk of GERD and EAC in patients with cholelithiasis or cholecystectomy (16, 20–26). The conflicting findings may arise from variations in study design, disease severity, and potential confounding factors. Therefore, it is difficult to distinguish whether cholelithiasis and cholecystectomy increase the risk of GERD, BE, or EAC.

The Mendelian randomization (MR) method is regarded as a natural randomized controlled trial (27), utilizing genetic variants that are strongly associated with the effects on outcomes (28). The MR method offers a valuable approach to enhance the robustness of causal inferences compared to conventional epidemiological research, particularly when addressing confounding factors and reverse causality (29). The meta-analysis is a comprehensive analytical method that summarizes the findings of cross-sectional or longitudinal observational studies. However, due to the inherent limitations of such studies, investigating the causal relationships of diseases becomes challenging. Therefore, meta-analysis and MR analysis complement each other in terms of the study design and inferring causality. The meta-analysis is suitable for providing a comprehensive evaluation of available evidence, while the MR analysis is more appropriate for making causal inferences. A more holistic perspective can be achieved by combining these two methods, leading to robust conclusions in scientific research. In this study, we performed a meta-analysis of observational studies to assess the association of cholelithiasis and cholecystectomy with the risk of GERD, BE, and EAC. We, subsequently, performed an MR analysis to assess the causal effects of cholelithiasis and cholecystectomy on the risk of GERD, BE, and EAC. The previously known risk factors for GERD, BE, and EAC were also considered in the multivariate MR analysis.

## Materials and methods

### Meta-analysis based on literature search

The meta-analysis followed the Meta-analysis of Observational Studies in Epidemiology (MOOSE) reporting guidelines (Supplementary Table S1). In November 2023, a comprehensive literature search was conducted across three prominent databases (PubMed, Embase, and Web of Science) using relevant medical subject terms and free keywords to cholelithiasis, cholecystectomy, esophageal diseases, GERD, BE, and EAC. Furthermore, we manually screened references from relevant studies to identify other eligible studies. The detailed search strategies are in the Supplementary Table S2.

### Inclusion and exclusion criteria

The studies eligible for the meta-analysis should (1) be conducted using a cohort study or a case–control design; (2) report the cholelithiasis and cholecystectomy as the exposure, and the incidences GERD, BE, and EAC as the outcome of interest; (3) present risk estimates as evaluated by odds ratio (OR), hazard ratio (HR), and relative risk (RR) along with their corresponding 95% Confidence Intervals (CIs), and (4) incorporate the possibility of a connection between cholelithiasis or cholecystectomy and GERD, BE, and EAC in the general population. We excluded studies with less than 20 participants, posters or meeting abstracts, editorials, case reports, reviews, guidelines, meta-analysis, non-observational studies, non-English studies, non-human studies, studies with no experimental or control data, studies on animal, or *in vitro* studies.

### Data extraction and quality assessment

Two reviewers (HY and MYC) extracted the data from Microsoft Excel for meta-analysis. The data contained the following information: first author, study location, publication date, study design, number of cases, number of controls/cohorts, exposure and outcome, and risk estimates. Two researchers independently utilized the Cochrane risk bias assessment tool to generate random distributions in the literature. They also allocated concealment, ensured blinding for both researchers and subjects, blinded the outcome indicators, assessed the completeness of outcome data, and evaluated selective reporting studies. The risk of bias was assessed by considering the results and the other potential sources of bias. In the cases where there was disagreement between the opinion of two parties, a third party’s perspective was sought and discussed to reach an agreement.

### Statistical analysis

The meta-analysis was performed using RevMan 5.4 software. In this meta-analysis, we used RR as a risk estimate to assess the association of cholelithiasis and cholecystectomy with GERD, BE, and EAC. If the outcome corresponded to only one exposure study, we did not perform the meta-analysis. The heterogeneity of the studies was assessed based on the P and I^2^ values. If the results of each study did not exhibit substantial statistical heterogeneity (*I*^2^ ≤ 50%; *p* ≥ 0.1), a meta-analysis was conducted utilizing a fixed-effects model. If there was a presence of statistical heterogeneity among the findings of each study (*I*^2^ > 50%; *p* < 0.1), it was necessary to conduct further investigation into the underlying causes of this heterogeneity. A random effects model was subsequently used for conducting the meta-analysis, and the test level of the meta-analysis was set to *p* = 0.05. The *p* values were two-sided.

### Mendelian randomization using GWAS data sources

We obtained summary statistics for the cholelithiasis and cholecystectomy genome-wide association study (GWAS) from FinnGen Biobank of European ancestry. There were 37,041 cases and 330,903 controls for cholelithiasis, and 26,778 cases and 350,499 controls for cholecystectomy.^1^ FinnGen is a large public-private partnership that aims to collect and analyze genomic and health data for 500,000 Finnish participants. The genetic data lists for GERD, BE, and EAC were obtained from the largest GWAS, and downloaded from its public website “open GWAS”.^2^ There were 129,080 cases and 602,604 controls for GERD, 13,358 cases and 56,429 controls for BE, and 740 cases and 372,756 controls for EAC. The waist-to-hip ratio for calculating the Body Mass Index (BMI) was determined using the GIANT consortium (30). Smoke, drink GWAS summary-level data were generated by GWAS & Sequencing Consortium of Alcohol and Nicotine Use (GSCAN) (31). Each GWAS was approved by its corresponding Ethics Committee and these data could be used without restriction.

### Single nucleotide polymorphism (SNP) selection

To validate the causal relationship between cholelithiasis, cholecystectomy, and the risk of GERD, BE, and EAC, the appropriate instrument variables were selected using quality control procedures. First, the SNPs significantly associated with cholelithiasis and cholecystectomy were chosen as instrumental variables (IVs) using thresholds (5 × 10^−8^). Second, we conducted linkage disequilibrium analysis across SNPs based on the reference panel from the European 1,000 Genomes Project (*r*^2^ < 0.01 and clump distance >10,000 kb). Third, if the minor allele frequency was less than 0.42 for each palindromic SNP, the SNP was judged inferable.

### Statistical analysis of the MR estimates

To ascertain that the effect of each IV on exposure and outcome corresponded with the same allele, we coordinated cholelithiasis, cholecystectomy, and GERD, BE, and EAC data. The MR analysis was performed using Inverse-variance weighted (IVW), weighted median, and MR-Egger methods for all the enrolled individuals. The IVW, as the main method, is the most accurate method while assuming that all the SNPs are available as valid variables (32). Furthermore, the weighted median approach yields consistent estimates assuming that more than half of the weights are from valid SNP (33). The MR-Egger analysis can calibrate for pleiotropy and calculate causal inferences, even when all genetic variants are pleiotropic (34).

**Figure 1:**
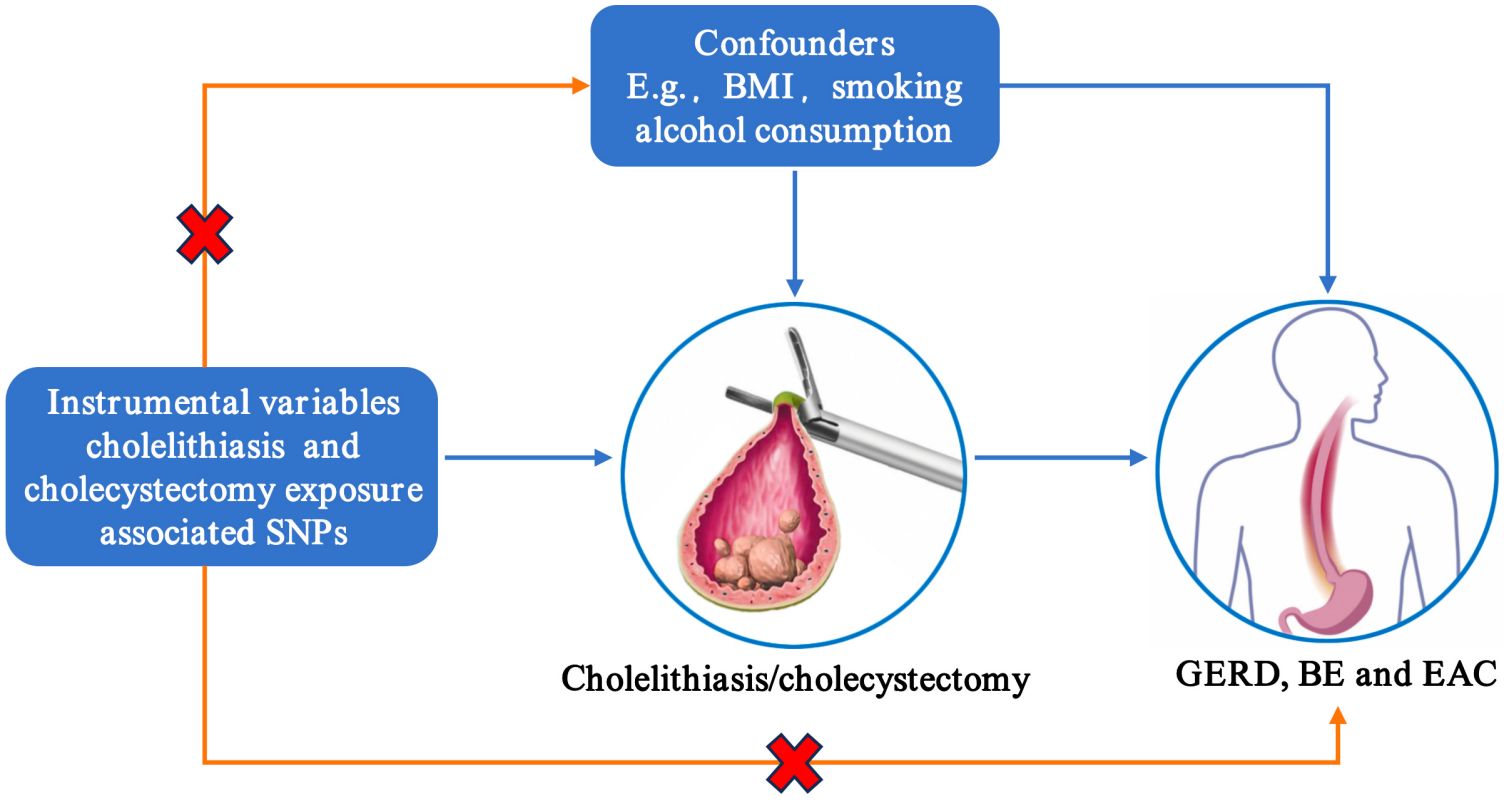
Assumptions of Mendelian randomization analysis. SNP, single-nucleotide polymorphism; BMI, body mass index; GERD, gastroesophageal reflux disease; BE, Barrett’s esophagus; EAC, esophageal adenocarcinoma.

The factors such as smoking, body mass index (BMI), and drinking have been associated with the risk of GERD, BE, and EAC (35). These risk factors may mediate the effect of gallstones on GERD, BE, and EAC. We used multivariable MR (MVMR) to assess the direct impact of cholelithiasis or cholecystectomy on GERD, BE, and EAC. Gallstones and all other risk factors with a causal effect on GERD, BE, and EAC risk were simultaneously considered in a multiway MVMR. The overview design of the current MR study is shown in Figure 1.

### Sensitivity analysis

Several essential sensitivity analyses were used to verify the results. A test for heterogeneity was conducted using Cochrane’s tool (36). The MR-Egger regression intercept could estimate the potential pleiotropy between the exposure and outcome. An intercept *p*-value of >0.05 suggested there was no horizontal pleiotropy (37). Furthermore, a leave-one-out analysis was also used to detect the pleiotropy caused by each SNP. All analyses were conducted by employing the two-sample MR and MVMR packages. The results were presented as odds ratios with 95% confidence intervals (CI). All the presented *p*-values were two-sided and statistical significance was set at 5%.

## Results

### Literature search results

**Figure 2:**
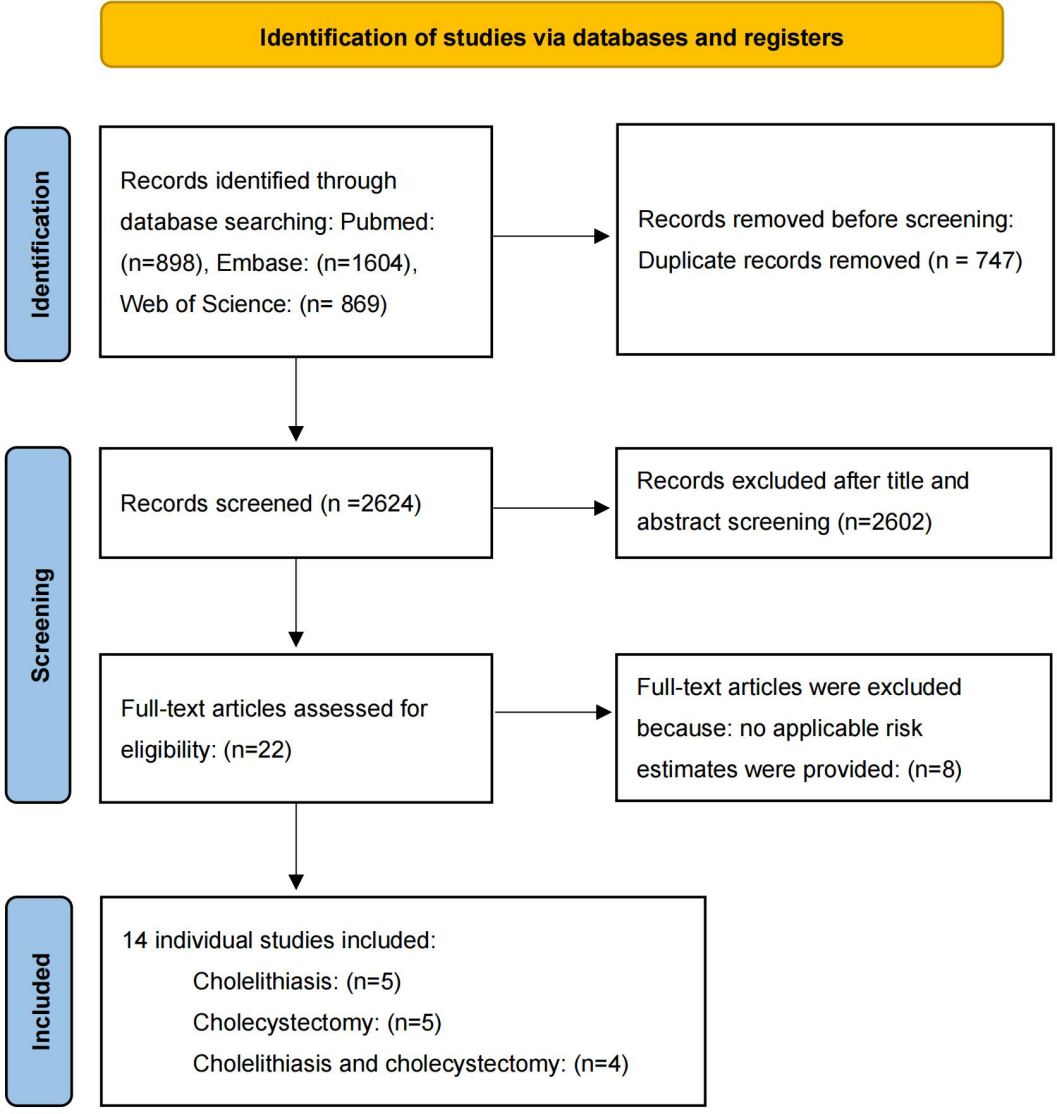
Flow chart of literature screening on meta-analysis.

After screening, five literatures on cholelithiasis, five on cholecystectomy, and four on cholelithiasis and cholecystectomy were included. The basic characteristics of the studies are shown in Table 1. The bias risk maps are included in the Supplementary Figures S1, S2. The PRISMA flow diagram of the meta-analysis is shown in Figure 2.

**Table 1:** Characteristics of studies included in the meta-analysis.

Authors, year	Study design	No. of cases	No. of controls	Exposure	Outcomes	Risk estimates
Chen (38)	Cohort	15,545	62,180	Cholelithiasis	EAC	HR: 0.92, 95%CI (0.50–1.70)
Freedman (24)	Case–control	92	728	Cholecystectomy	EAC	OR: 1.03, 95%CI (0.63–1.69)
Freedman (16)	Cohort	268,312	250,087	Cholecystectomy	EAC	RR: 1.3, 95%CI (1.0–1.8)
Izbéki (15)	Case–control	203	504	Cholelithiasis	BE	OR:2.08, 95%CI (1.44–2.99)
Lagergren (17)	Cohort	345,251	342,867	Cholecystectomy	EAC	RR:1.29, 95%CI (1.07–1.53)
Lagergren (17)	Cohort	199,960	201,495	Cholelithiasis	EAC	RR: 0.99, 95%CI (0.71–1.35)
Matsu (14)	Case–control	528	528	Cholelithiasis	BE	OR: 1.56, 95%CI (1.09–2.25)
Nogueira (39)	Case–control	5,488	100,000	Cholecystectomy	EAC	OR: 0.95, 95%CI (0.80–1.14)
Nogueira (39)	Case–control	5,488	100,000	Cholelithiasis	EAC	OR: 1.05, 95%CI (0.91–1.21)
Shabanzadeh (40)	Cohort	129,484	4,442,528	Cholecystectomy	EAC	HR: 0.57, 95%CI (0.43–0.74)
Shabanzadeh (40)	Cohort	146,216	4,442,528	Cholelithiasis	EAC	HR: 0.93, 95%CI (0.67–1.29)
Shabanzadeh (21)	Cohort	591	5,337	Cholelithiasis	EAC	HR: 0.66, 95%CI (0.20–2.19)
Tavani (22)	Case–control	917	3,666	Cholelithiasis	EAC	OR: 1.15, 95%CI (0.80–1.65)
Zhao (41)	Cohort	1857	77,804	Cholelithiasis	EAC	OR: 0.59, 95%CI (0.14–2.37)
Zhao (41)	Cohort	148	77,804	Cholecystectomy	EAC	OR: 1.96, 95%CI (0.12–31.63)
Goldacre (42)	Cohort	39,254	334,813	Cholecystectomy	EAC	RR: 0.98, 95%CI (0.79–1.21)
Doyle (43)	Cohort	20	17	Cholecystectomy	GERD	OR: 6.30, 95%CI (1.09–36.30)
McNamara (44)	Cohort	212	62	Cholecystectomy	GERD	OR: 1.99, 95%CI (1.12–3.52)

GERD, gastroesophageal reflux disease; BE, Barrett’s esophagus; EAC, esophageal adenocarcinoma; OR, odds ratio; HR, hazard ratio; RR, relative risk.

### Observed associations between cholelithiasis and risk of GERD, BE, and EAC

**Figure 3:**
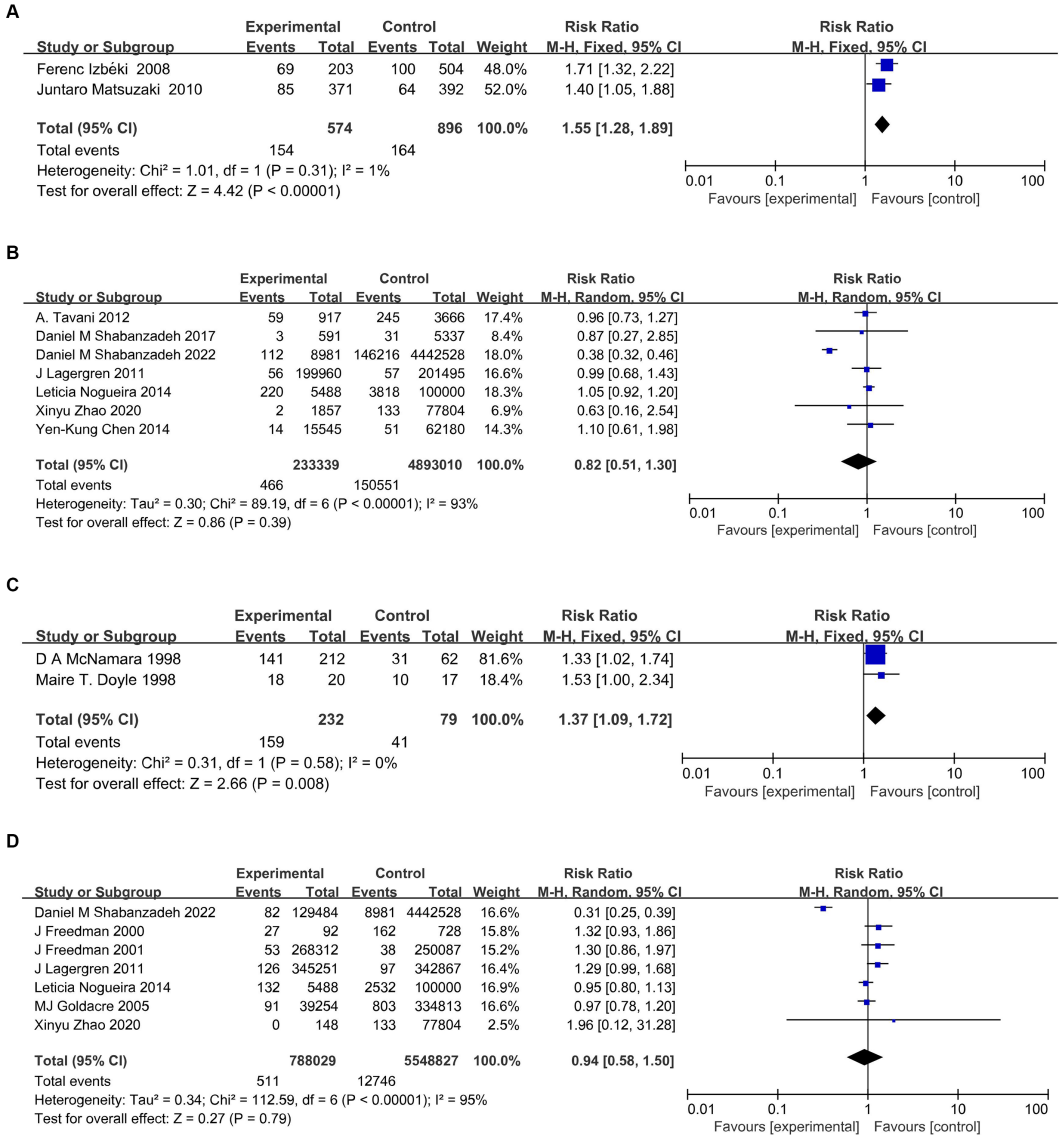
Meta-analysis on the relationship of cholelithiasis with BE **(A)**, and EAC **(B)**, and of cholecystectomy with GERD **(C)**, and EAC **(D)**. BE, Barrett’s esophagus; GERD, gastroesophageal reflux disease; EAC, esophageal adenocarcinoma.

Cholelithiasis was associated with BE in two independent studies (14, 15). The pooled RR of BE was 1.77 (95% CI, 1.37–2.29; *p* < 0.001). Among the seven studies evaluating the association between cholelithiasis and risk of EAC, two studies demonstrated an elevated risk of EAC; however, the pooled results were not statistically significant (38, 39). This indicated that insufficient research had been conducted to examine the correlation between cholelithiasis and GERD (Figures 3A,B).

### Observed associations between cholecystectomy and GERD, BE, and EAC risk

Cholecystectomy was linked to GERD in two independent studies (RR, 1.37; 95%CI, 1.09–1.72; *p* = 0.008) (43, 44). There was not enough data to conduct a meta-analysis of cholecystectomy and BE. Cholecystectomy was identified as a significant risk factor for EAC in four out of the seven studies. However, the pooled studies were not statistically significant (16, 17, 24, 41). Furthermore, heterogeneity was not detected in all analyses in the studies (Figures 3C,D).

### Genetic association between cholelithiasis and GERD, BE, and EAC

**Figure 4:**
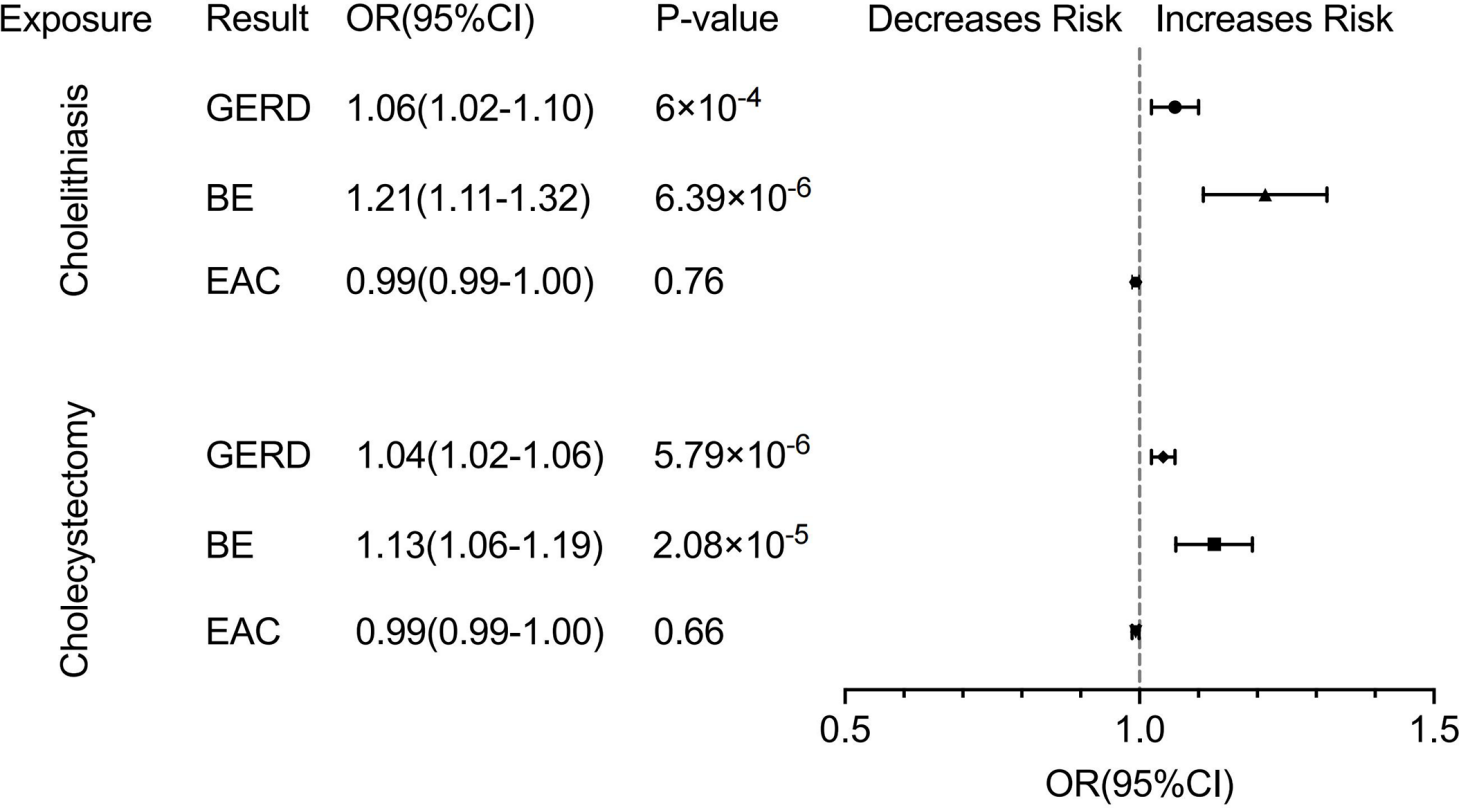
Forest plot of univariate Mendelian randomization analysis. GERD, gastroesophageal reflux disease; BE, Barrett’s esophagus; EAC, esophageal adenocarcinoma.

We observed a robust correlation in our primary analysis of cholelithiasis and its association with GERD, BE, and EAC. Cholelithiasis exhibited a significant association with an increased risk of both GERD (OR, 1.06; 95% CI,1.02–1.10; *p* < 0.001) and BE (OR, 1.21; 95% CI, 1.11–1.32; *p* = 6.39 × 10^−6^) according to the IVW methods. The results of the Weighted median and MR-Egger methods are consistent with those of IVW methods (GERD: OR, 1.05; 95% CI, 1.02–1.09 for the weighted median methods, OR, 1.06; 95% CI, 0.97–1.16 for the MR-Egger methods. BE: OR, 1.20; 95% CI, 1.08–1.33 for the weighted median methods, OR, 1.24; 95% CI, 1.01–1.51 for the MR-Egger methods). The results obtained from the MR-Egger methods on GERD did not indicate statistical significance. However, the observed trend aligned with the findings of the primary analysis. We did not find a causal relationship between cholelithiasis and EAC (Figure 4; Supplementary Table S3). The forest, leave-one-out, and scatter plots were presented in Supplementary Figure S3. Furthermore, horizontal pleiotropy was not found in the MR results (Supplementary Table S3).

### Genetic association between cholecystectomy and GERD, BE and EAC

In the analysis of cholecystectomy and its association with GERD, BE, and EAC, we also observed a significant correlation between cholecystectomy and an increased risk of developing GERD (OR, 1.04; 95% CI, 1.02–1.06; *p* = 5.79 × 10^−6^ for the IVW methods, OR, 1.04; 95% CI, 1.02–1.06 for the weighted median methods, OR, 1.04; 95% CI, 1.00–1.07 for the MR-Egger methods) and BE (OR, 1.13; 95% CI, 1.06–1.19; *p* = 2.08 × 10^−5^ for the IVW methods, OR, 1.08; 95% CI, 1.01–1.15 for the weighted median methods, OR, 1.09; 95% CI, 0.99–1.19 for the MR-Egger methods). Furthermore, our findings did not indicate an increased risk of EAC associated with cholecystectomy, as in the case of cholelithiasis (Figure 4; Supplementary Table S3). The forest, leave-one-out, and scatter plots were presented in Supplementary Figure S4. The MR results did not show any horizontal pleiotropy (Supplementary Table S3).

### Multivariable MR analysis adjusting potential confounders

In the multivariate analysis, we adjusted for common risk factors (smoking, alcohol consumption, BMI) which are commonly associated with GERD, BE, and EAC. The findings remained consistent with those observed in the univariate analysis. Positive genetic relationships of cholelithiasis with GERD (OR, 1.05; 95% CI, 1.01–1.10; *p* = 0.028) and BE (OR, 1.18; 95% CI,1.07–1.31; *p* = 0.002) and cholecystectomy on GERD (OR, 1.07; 95% CI, 1.02–1.12; *p* = 0.002) and BE (OR, 1.18, 95% CI, 1.05–1.33; *p* = 0.005) were found (Supplementary Table S4).

## Discussion

This is the first time that a meta-analysis and MR analysis have been used to assess the causal relationship between cholelithiasis, cholecystectomy, and the risk of GERD, BE, and EAC. Cholelithiasis has been identified as a significant risk factor for BE in our studies, while cholecystectomy is associated with an increased risk of GERD. Although several studies have provided evidence for the association between cholelithiasis or cholecystectomy and an increased risk of EAC, the pooled odds ratios exhibit no statistical significance. Considering the limited number of studies and the absence of a meta-analysis on the association between cholelithiasis and GERD, and between cholecystectomy and BE, we conducted a comprehensive investigation into the causal association between cholelithiasis, cholecystectomy, and the three esophageal disorders, using MR analysis. We found that cholelithiasis and cholecystectomy may serve as casual risk factors for GERD and BE development. Our MR analysis did not reveal a causal association between cholelithiasis or cholecystectomy and EAC, this was consistent with our meta-analysis results. To better understand the associations of cholelithiasis and cholecystectomy among GERD and BE, we performed a multivariate MR analysis incorporating the known risk factors for GERD and BE. After adjusting for confounding factors including smoking, alcohol consumption, and BMI, cholelithiasis or cholecystectomy remained the significant risk factors for GERD and BE.

In an empty stomach, bile is usually stored in the gallbladder and flows to the duodenum under the influence of cholecystokinin (CCK), an enteral hormone (13, 45, 46). The bile storage site is not present after cholecystectomy. However, bile continues to be secreted, significantly increasing the likelihood of its regurgitation into the duodenum, stomach, and esophagus (9). In the interim, elevated serum cholecystokinin (CCK) levels were observed following cholecystectomy. The enteral hormone CCK is ordinarily inhibited by negative feedback from bile (47, 48). This regulatory mechanism gets impaired, following cholecystectomy, resulting in a persistent elevation of CCK levels (46). An increase in circulating CCK triggers a decline in lower esophageal sphincter pressure (49) and an escalation in the frequency of transient lower esophageal sphincter relaxation episodes (TLESREs), further exacerbating the exposure of the esophageal mucosa (50). There seems to be a clear causal relationship between cholecystectomy and esophageal bile reflux (45, 46). There are studies supporting the relationship between cholelithiasis and bile reflux (51). The gallbladder contractions effectively result in the regular excretion of tiny cholesterol crystals, thereby preventing stone formation. Therefore, the generation of cholelithiasis has a certain relationship with impaired gallbladder movement. Studies have reported that gallbladder dysfunction or non-function is associated with continued bile flow into the duodenum, stomach, and esophagus (52, 53). According to the literature, studies on animal models have reported that surgically induced bile reflux can lead to the development of severe GERD (54–56). Since GERD is a major cause of BE, which in turn is a major risk factor for EAC (6), it seems reasonable that there is an increased risk of GERD, BE, and EAC after cholecystectomy and cholelithiasis.

How does bile work on the esophageal epithelium? Through the current research, there are several hypotheses. First, using animal models and human studies, research demonstrates that esophageal mucosal damage can result from the synergistic interactions between bile acids and stomach acids, as well as the synergistic interaction of bile acids and trypsin at a more neutral pH (57, 58). Second, bile can activate COX-2 transcription in EAC cell lines, which through a signaling cascade (protein kinase C(PKC)-phosphatidylinositol-3 kinase (PI-3 K)-mitogen-activated protein kinase (ERKI/MAPK)) increases cell proliferation (59–61). Third, bile reflux induces alterations in the esophageal microbiota (62, 63), wherein the bacteria enzymatically converts primary bile acids into secondary bile acids through dihydroxylation (64). These secondary bile acids can subsequently trigger DNA damage, thereby facilitating the development of atypical hyperplasia in esophageal epithelial tissue (65). These findings help to explain the mechanism by which bile promotes GERD, BE, and EAC, but they still need to be explored.

Although the relationship between cholelithiasis or cholecystectomy on GERD, BE and EAC has not been a consensus in clinical studies, our MR analysis results provide some support for the observation that cholelithiasis or cholecystectomy increases the risk of GERD and BE (14, 18). We observe that cholelithiasis and cholecystectomy emerge as robust risk factors for GERD and BE, considering the outcomes in observational studies and the results of MR analysis. We did not find relationship between cholelithiasis or cholecystectomy and EAC, in both observational studies and MR analysis. However, considering reflux as a significant risk factor for EAC (3), and the causal relationship between cholelithiasis or cholecystectomy and GERD or BE, we postulate that these findings could be attributed to the fact that only a minor proportion of GERD and BE progress to EAC under clinical management, while symptomatic cholelithiasis receives standard treatment (66). Additionally, it is possible that the development of EAC may be more influenced by other factors not directly associated with bile. However, we maintain that cholelithiasis and cholecystectomy might exert certain influences on the initial stages of esophageal reflux disease.

The advantages of this meta-analysis and the MR analysis are: This study represents the first meta-analysis investigating the contentious association of cholelithiasis and cholecystectomy with an increased risk of GERD, BE, and EAC by discussing bile reflux, as noted in the observational studies. In addition, we used the MR analysis to investigate the causal relationship between cholelithiasis, cholecystectomy, and GERD, BE, and EAC from a genetic perspective. Furthermore, the meta-analysis considered high-quality literature, with comprehensive and up-to-date data, encompassing the most recent publications. Finally, in the MR analysis, we used GERD and BE data from the same cohort, which increase the reliability of the analysis. However, this study has certain limitations: First, in the MR analysis, our study does not explain the mechanism by which bile reflux leads to esophageal metaplasia (BE) and malignant transformation (EAC). Second, in the MR analysis, our sample was of European ancestry, which limits generalizations to other lineages. Third, it should be noted that the meta-analysis may introduce certain biases due to variations in population characteristics and study quality. We placed particular emphasis on the inclusion of high-quality studies to consider the bias in meta-analysis. We conducted a sensitivity analysis in the MR analysis to improve the reliability of our findings. Furthermore, we planned to replicate the study in diverse populations, thereby augmenting both validity and generalizability of the results.

## Conclusion

Our findings suggest that cholelithiasis may increase the risk of GERD and BE, regardless of cholecystectomy. According to our study, we recommend early monitoring of esophageal symptoms in cholelithiasis patients, regardless of whether they have undergone cholecystectomy. Furthermore, prompt treatment should be initiated upon the occurrence of GERD or BE to mitigate disease progression. Our finding proposed that invasive EAC cytology is not deemed necessary in these patients until esophageal metaplasia has been manifested. Finally, further work is warranted to decipher the underlying mechanisms linking esophageal disorder and bile reflux.

## Data availability statement

The datasets presented in this study can be found in online repositories. The names of the repository/repositories and accession number(s) can be found at: We obtained summary statistics for the cholelithiasis and cholecystectomy genome-wide association study (GWAS) from FinnGen Biobank of European ancestry: https://www.finngen.fi/en/access_results. The genetic data lists for GERD, BE, and EAC were acquired from the largest GWAS and downloaded from its public website “open GWAS” (https://gwas.mrcieu.ac.uk/).

## Author contributions

YH: Methodology, Writing – original draft. YCa: Writing – review & editing, Supervision. YCh: Validation, Writing – review & editing. QZ: Writing – review & editing. WF: Writing – original draft. LJ: Writing – review & editing. YM: Data curation, Writing – review & editing.

## Footnotes

^1^ https://storage.googleapis.com/finngen-public-data-r9/summary_stats/finngen_R9_K11_CHOLELITH.gz

^2^ https://storage.googleapis.com/finngen-public-data-r9/summary_stats/finngen_R9_K11_CHOLECYSTECTOMY.gz
