# Supplementary material for: Cholelithiasis and cholecystectomy increase the risk of gastroesophageal reflux disease and Barrett’s esophagus
Source: Front Med (Lausanne). 2024 Jul 18;11:1420462. doi: 10.3389/fmed.2024.1420462 (PMC11292949; doi:10.3389/fmed.2024.1420462)
Supplement: Supplementary file 1 [file Data_Sheet_1.docx]

Supplementary Material

# Supplementary Figures and Tables

## Supplementary Figures


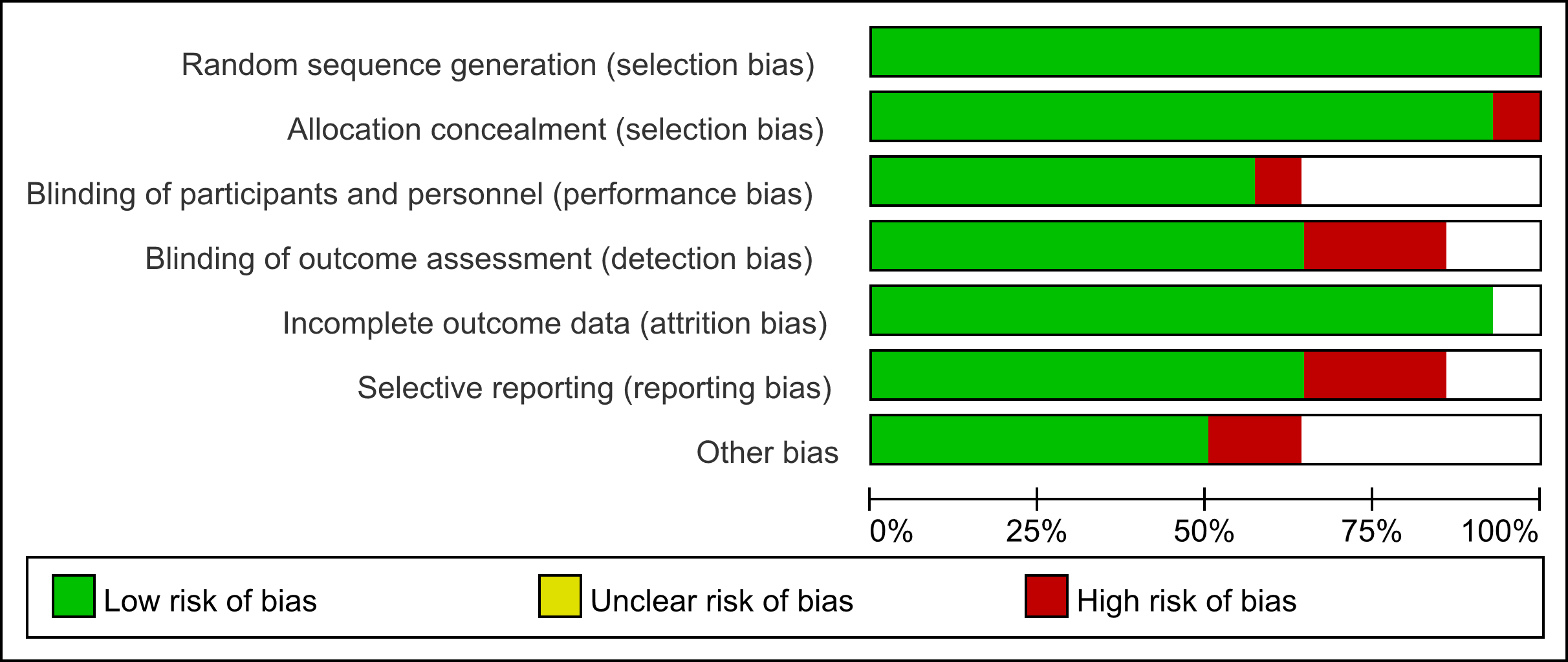


**Supplementary Figure S1:** Risk of bias assessment tool for included studies for the Meta-analysis.


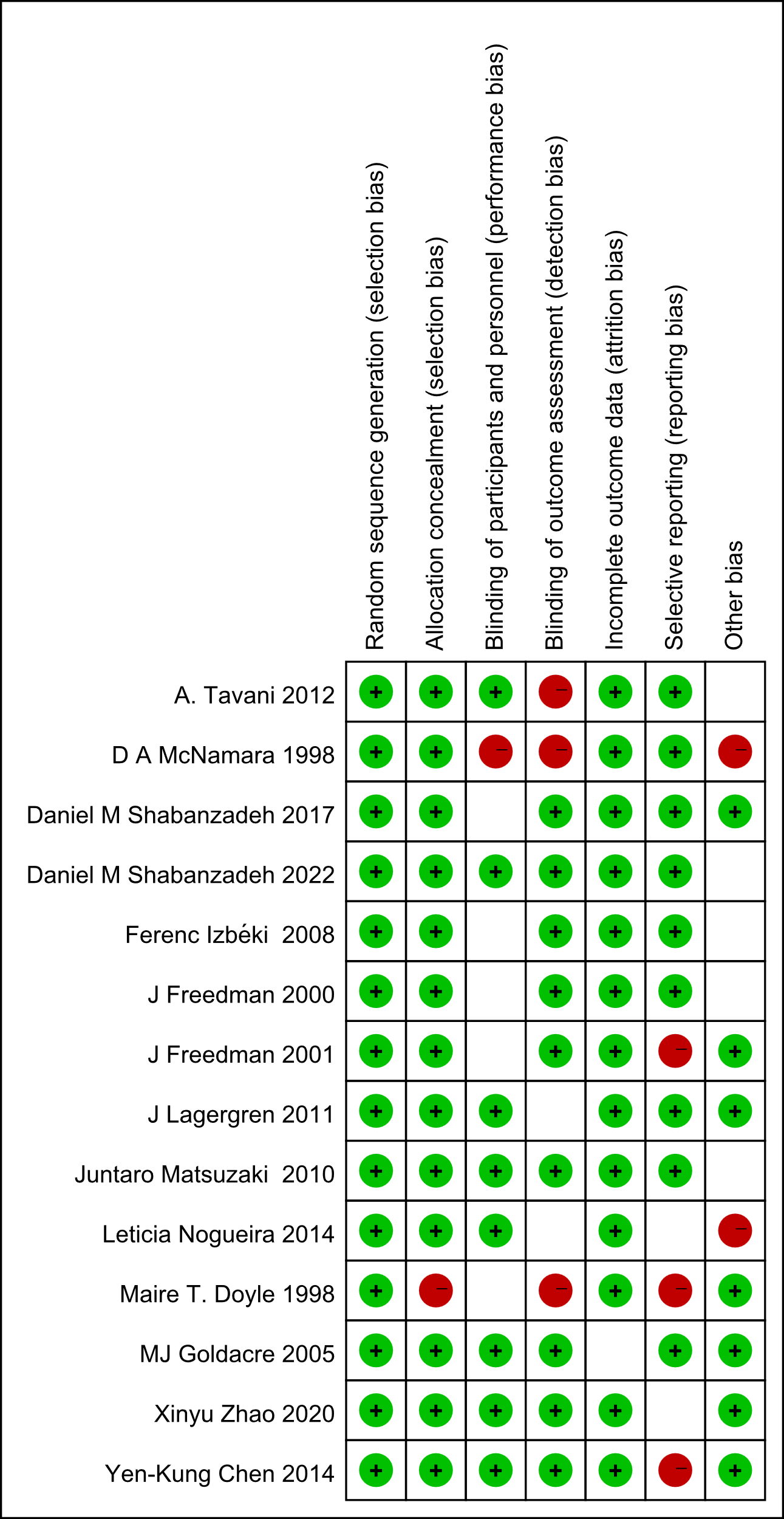


**Supplementary Figure S2:** Summary of risk of bias in included studies for the Meta-analysis.


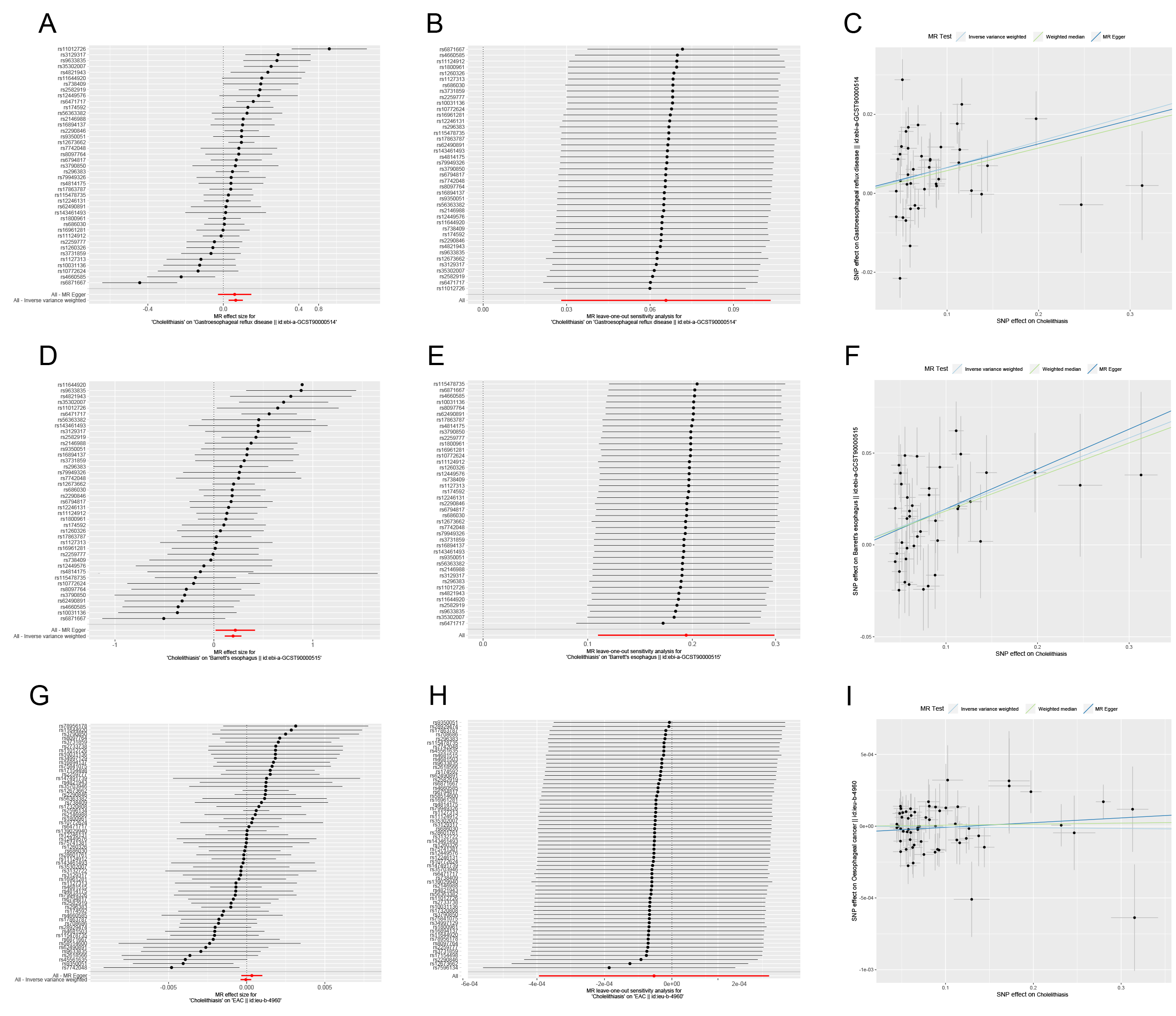


**Supplementary Figure S3:** Forest plot between cholelithiasis and GERD (A), BE (D), and EAC (G). Leave-one-out analysis between GERD (B), BE (E), and EAC (H). Scatter plot between GERD (C), BE (F), EAC (I).

**Abbreviation:** GERD, gastroesophageal reflux disease; BE, Barrett's esophagus; EAC, esophageal adenocarcinoma.


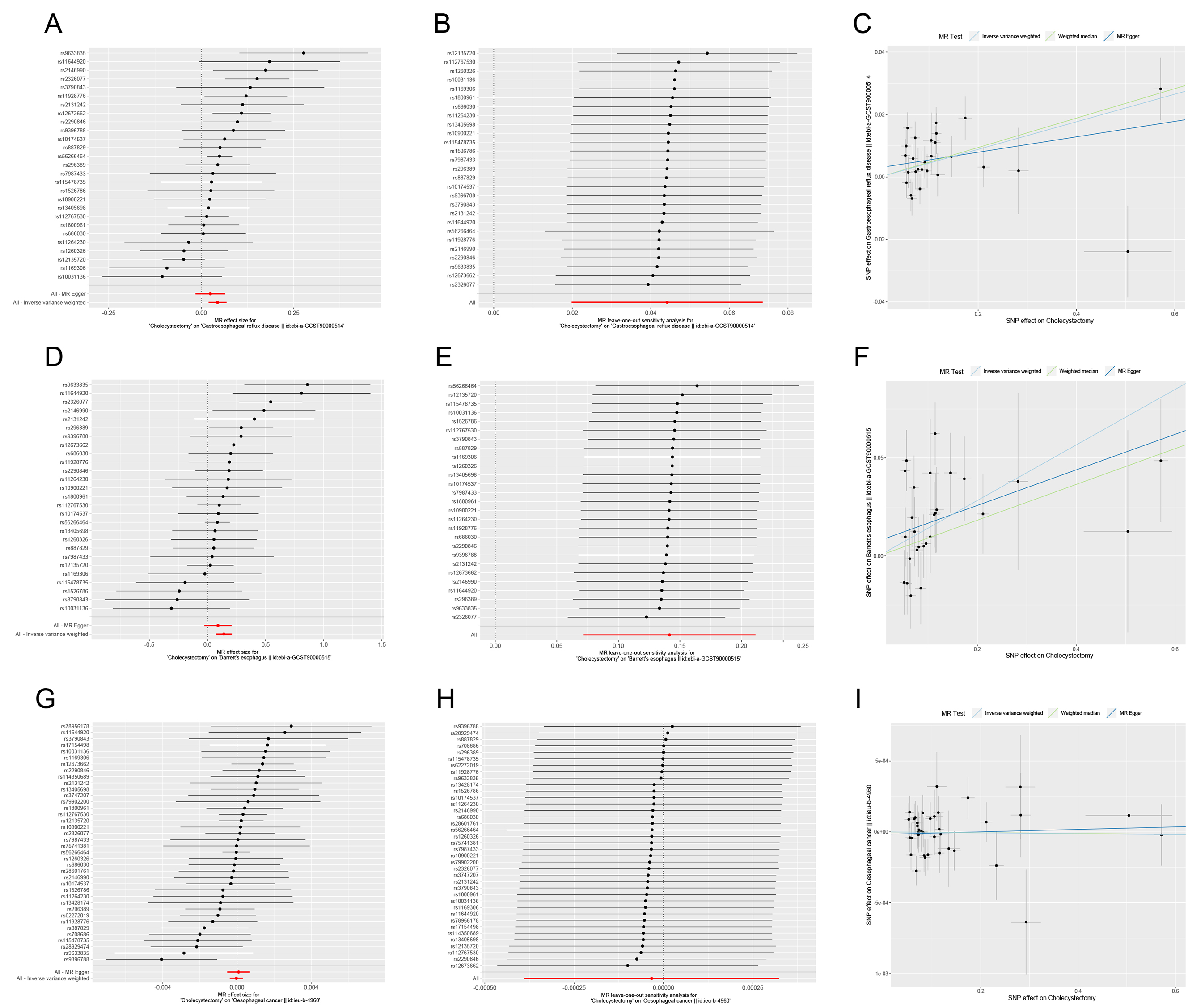


**Supplementary Figure S4:** Forest plot between cholecystectomy and GERD (A), BE (D), and EAC (G). Leave-one-out analysis between GERD (B), BE (E), and EAC (H). Scatter plot between GERD (C), BE (F), EAC (I).

**Abbreviation:** GERD, gastroesophageal reflux disease; BE, Barrett's esophagus; EAC, esophageal adenocarcinoma.

## Supplementary Tables

| Item No | Recommendation | Reported on Page No |
| --- | --- | --- |
| Reporting of background should include | | |
| 1 | Problem definition | Background paragraph 2 |
| 2 | Hypothesis statement | Background paragraph 2 |
| 3 | Description of study outcome(s) | Result paragraph 2 and 3 |
| 4 | Type of exposure or intervention used | Methods paragraph 1 |
| 5 | Type of study designs used | Methods paragraph 1 |
| 6 | Study population | Methods paragraph 2 |
| Reporting of search strategy should include | | |
| 7 | Qualifications of searchers (eg,librarians and investigators) | Methods paragraph 3 |
| 8 | Search strategy, including time period included in the synthesis and key words | Supplement |
| 9 | Effort to include all available studies, including contact with authors | Supplement |
| 10 | Databases and registries searched | NA |
| 11 | Search software used,name and version, including special features used (eg,explosion) | NA |
| 12 | Use of hand searching (eg,reference lists of obtained articles) | Methods paragraph 3 |
| 13 | List of citations located and those excluded, including justification | Methods paragraph 3 |
| 14 | Method of addressing articles published in languages other than English | NA |
| 15 | Method of handling abstracts and unpublished studies | Supplement |
| 16 | Description of any contact with authors | Supplement |
| Reporting of methods should include | | |
| 17 | Description of relevance or appropriateness of studies assembled for assessing the hypothesis to be tested | Methods paragraph 3 |
| 18 | Rationale for the selection and coding of data (eg,sound clinical principles or convenience) | NA |
| 19 | Documentation of how data were classified and coded (eg,multiple raters,blinding anonterrater reliability) | NA |
| 20 | Assessment of confounding (eg,comparability of cases and controls in studies where appropriate) | Methods paragraph 3 |
| 21 | Assessment of study quality, including blinding of quality assessors, stratification or regression on possible predictors of study results | Methods paragraph 3 |
| 22 | Assessment of heterogeneity | Supplement |
| 23 | Description of statistical methods (eg,complete description of fixed or random effects models, justification of whether the chosen models account for predictors of study results,dose-response models,or cumulative meta-analysis)in sufficient detail to be replicated | Methods paragraph 4 |
| 24 | Provision of appropriate tables and graphics | Supplement |
| Reporting of results should include | | |
| 25 | Graphic summarizing individual study estimates and overall estimate | Figure4 |
| 26 | Table giving descriptive information for each study included | Table1 |
| 27 | Results of sensitivity testing (eg,subgroup analysis) | Result paragraph 2 and 3 |
| 28 | ndication of statistical uncertainty of findings | NA |

**Supplementary Table S1:** MOOSE Checklist for Meta-analyses of Observational Studies

| **Database** | **Search strategies** | **Results** |
| --- | --- | --- |
| PubMed | ((("Cholelithiasis"[Mesh]) OR (Cholelithiasis) OR ("Cholelithiases"[MeSH Terms]) OR ("Cholelithiases") OR ("Gallstone Disease"[MeSH Terms]) OR ("Gallstone Disease") OR ("Gallstone Diseases"[MeSH Terms]) OR ("Gallstone Diseases") OR ("Cholecystectomy"[Mesh]) OR (Cholecystectomy) OR (Cholecystectomies[MeSH Terms]) OR (Cholecystectomies)) AND (("Barrett Esophagus"[Mesh]) OR ("Barrett Esophagus") OR ("Barrett Metaplasia" or "Barrett Metaplasias" or "Metaplasia, Barrett" or "Metaplasias, Barrett" or "Barrett's Syndrome" or "Barretts Syndrome" or "Barrett Syndrome" or "Barrett's Esophagus" or "Barretts Esophagus" or "Esophagus, Barrett's" or "Esophagus, Barrett" or "Barrett Epithelium" or "Epithelium, Barrett") OR ("Esophageal Diseases"[Mesh]) OR ("Esophageal Diseases") OR ("Disease, Esophageal"[MeSH Terms]) OR ("Disease, Esophageal") OR ("Diseases, Esophageal"[MeSH Terms]) OR ("Diseases, Esophageal") OR ("Esophageal Disease"[MeSH Terms]) OR ("Esophageal Disease") OR (esophageal or esophagus) OR ("Gastroesophageal Reflux"[Mesh]) OR ("Gastroesophageal Reflux") OR ("Gastric Acid Reflux" or "Acid Reflux, Gastric" or "Reflux, Gastric Acid" or "Gastric Acid Reflux Disease" or "Gastro-Esophageal Reflux Disease" or "Gastro Esophageal Reflux Disease" or "Gastro-Esophageal Reflux Diseases" or "Reflux Disease, Gastro-Esophageal" or "Gastro-oesophageal Reflux" or "Gastro oesophageal Reflux" or "Reflux, Gastro-oesophageal" or "Gastroesophageal Reflux Disease" or "GERD" or "Reflux, Gastroesophageal" or "Esophageal Reflux" or "Gastro-Esophageal Reflux" or "Gastro Esophageal Reflux" or "Reflux, Gastro-Esophagea") OR ("Esophageal Neoplasms"[Mesh]) OR ("Esophageal Neoplasms") OR ("Esophageal Neoplasm" or "Neoplasm, Esophageal" or "Esophagus Neoplasm" or "Esophagus Neoplasms" or "Neoplasm, Esophagus" or "Neoplasms, Esophagus" or "Neoplasms, Esophageal" or "Cancer of Esophagus" or "Cancer of the Esophagus" or "Esophagus Cancer" or "Cancer, Esophagus" or "Cancers, Esophagus" or "Esophagus Cancers" or "Esophageal Cancer" or "Cancer, Esophageal" or "Cancers, Esophageal" or "Esophageal Cancers") OR ("esophageal adenocarcinoma"))) | 898 records |
| Embase  Web of Science | ('cholelithiasis'/exp OR 'cholelithiasis' OR 'cholelithiases' OR 'gallstone disease'/exp OR 'gallstone disease' OR 'gallstone diseases' OR 'cholecystectomy'/exp OR 'cholecystectomy' OR 'cholecystectomies') AND ('barrett esophagus'/exp OR 'barrett esophagus' OR 'barrett metaplasia'/exp OR 'barrett metaplasia' OR 'barrett metaplasias' OR 'metaplasia, barrett' OR 'metaplasias, barrett' OR 'barretts syndrome' OR 'barrett syndrome'/exp OR 'barrett syndrome' OR 'barretts esophagus'/exp OR 'barretts esophagus' OR 'esophagus, barretts' OR 'esophagus, barrett' OR 'barrett epithelium' OR 'epithelium, barrett' OR 'esophageal diseases'/exp OR 'esophageal diseases' OR 'disease, esophageal' OR 'diseases, esophageal' OR 'esophageal disease'/exp OR 'esophageal disease' OR 'esophageal' OR 'esophagus'/exp OR 'esophagus' OR 'gastroesophageal reflux'/exp OR 'gastroesophageal reflux' OR 'gastric acid reflux' OR 'acid reflux, gastric' OR 'reflux, gastric acid' OR 'gastric acid reflux disease' OR 'gastro-esophageal reflux disease' OR 'gastro esophageal reflux disease' OR 'gastro-esophageal reflux diseases' OR 'reflux disease, gastro-esophageal' OR 'gastro-oesophageal reflux'/exp OR 'gastro-oesophageal reflux' OR 'gastro oesophageal reflux'/exp OR 'gastro oesophageal reflux' OR 'reflux, gastro-oesophageal' OR 'gastroesophageal reflux disease'/exp OR 'gastroesophageal reflux disease' OR 'gerd' OR 'reflux, gastroesophageal'/exp OR 'reflux, gastroesophageal' OR 'esophageal reflux'/exp OR 'esophageal reflux' OR 'gastro-esophageal reflux'/exp OR 'gastro-esophageal reflux' OR 'gastro esophageal reflux'/exp OR 'gastro esophageal reflux' OR 'reflux, gastro-esophagea' OR 'esophageal neoplasms'/exp OR 'esophageal neoplasms' OR 'esophageal neoplasm' OR 'neoplasm, esophageal' OR 'esophagus neoplasm'/exp OR 'esophagus neoplasm' OR 'esophagus neoplasms'/exp OR 'esophagus neoplasms' OR 'neoplasm, esophagus' OR 'neoplasms, esophagus' OR 'neoplasms, esophageal' OR 'cancer of esophagus' OR 'cancer of the esophagus'/exp OR 'cancer of the esophagus' OR 'esophagus cancer'/exp OR 'esophagus cancer' OR 'cancer, esophagus'/exp OR 'cancer, esophagus' OR 'cancers, esophagus' OR 'esophagus cancers' OR 'esophageal cancer'/exp OR 'esophageal cancer' OR 'cancer, esophageal' OR 'cancers, esophageal' OR 'esophageal cancers' OR 'esophageal adenocarcinoma'/exp)  TS=(“Cholelithiasis” OR "Cholelithiases" OR "Gallstone Disease" OR "Gallstone Diseases" OR “Cholecystectomy” OR “Cholecystectomies”) AND TS= ("Barrett Esophagus" OR "Barrett Metaplasia" OR "Barrett Metaplasias" OR "Metaplasia, Barrett" OR "Metaplasias, Barrett" OR "Barrett's Syndrome" OR "Barretts Syndrome" OR "Barrett Syndrome" OR "Barrett's Esophagus" OR "Barretts Esophagus" OR "Esophagus, Barrett's" OR "Esophagus, Barrett" OR "Barrett Epithelium" OR "Epithelium, Barrett" OR "Esophageal Diseases" OR "Disease, Esophageal" OR "Diseases, Esophageal" OR "Esophageal Disease" OR “esophageal” OR “esophagus” OR "Gastroesophageal Reflux" OR "Gastric Acid Reflux" OR "Acid Reflux, Gastric" OR "Reflux, Gastric Acid" OR "Gastric Acid Reflux Disease" OR "Gastro-Esophageal Reflux Disease" OR "Gastro Esophageal Reflux Disease" OR "Gastro-Esophageal Reflux Diseases" OR "Reflux Disease, Gastro-Esophageal" OR "Gastro-oesophageal Reflux" OR "Gastrooesophageal Reflux" OR "Reflux, Gastro-oesophageal" OR "Gastroesophageal Reflux Disease" OR "GERD" OR "Reflux,Gastroesophageal" OR "Esophageal Reflux" OR "Gastro-Esophageal Reflux" OR "Gastro Esophageal Reflux" OR "Reflux, Gastro-Esophagea" OR "Esophageal Neoplasms" OR "Esophageal Neoplasm" OR "Neoplasm, Esophageal" OR "Esophagus Neoplasm" OR "Esophagus Neoplasms" OR"Esophagus Cancer" OR "Cancer, Esophagus" OR "Cancers, Esophagus" OR "Esophagus Cancers" OR "Esophageal Cancer" OR "Cancer, Esophageal" OR "Cancers, Esophageal" OR "Esophageal Cancers" OR "esophageal adenocarcinoma") | 1604 records  869 records |

**Supplementary Table S2:** Search Strategy for the Meta-analysis

| **Exposure** | **Outcome** | **Methods** | **OR** | **P** | **Q-statistics** | **Ph** | **Egger**  **intercept** | **P intercept** |
| --- | --- | --- | --- | --- | --- | --- | --- | --- |
| Cholelithiasis | GERD | Inverse variance weighted | 1.06(1.02-1.10) | 0.0006 | 124.552 | 1.30×10^-10^ |  |  |
|  | GERD | MR Egger | 1.06(0.97-1.16) | 0.19 | 124.491 | 7.32×10^-11^ | 0.0005 | 0.891 |
|  | GERD | Weighted median | 1.05(1.02-1.09) | 0.001 |  |  |  |  |
|  | BE | Inverse variance weighted | 1.21(1.11-1.32) | 6.39×10^-6^ | 63.43 | 0.01 |  |  |
|  | BE | MR Egger | 1.24(1.01-1.51) | 0.039 | 63．331 | 0.008 | 0.002 | 0.806 |
|  | BE | Weighted median | 1.20(1.08-1.33) | 0.0003 |  |  |  |  |
|  | EAC | Inverse variance weighted | 0.99(0.99-1.00) | 0.76 | 59.78 | 0.484 |  |  |
|  | EAC | MR Egger | 1.00(0.99-1.00) | 0.34 | 58.106 | 0.508 | 4.13×10^-5^ | 0.2 |
|  | EAC | Weighted median | 1.00(0.99-1.00) | 0.79 |  |  |  |  |
|  | | | | | | | | |
| Cholecystectomy | GERD | Inverse variance weighted | 1.04(1.02-1.06) | 5.79×10^-6^ | 47.156 | 0.006 |  |  |
|  | GERD | MR Egger | 1.04(1.00-1.07) | 0.02 | 44.628 | 0.009 | 0.003 | 0.245 |
|  | GERD | Weighted median | 1.04(1.02-1.06) | 8.74×10^-5^ |  |  |  |  |
|  | BE | Inverse variance weighted | 1.13(1.06-1.19) | 2.08×10^-5^ | 38.553 | 0.053 |  |  |
|  | BE | MR Egger | 1.09(0.99-1.19) | 0.07 | 36.881 | 0.059 | 0.008 | 0.297 |
|  | BE | Weighted median | 1.08(1.01-1.15) | 0.01 |  |  |  |  |
|  | EAC | Inverse variance weighted | 0.99(0.99-1.00) | 0.66 | 35.211 | 0.553 |  |  |
|  | EAC | MR Egger | 1.00(0.99-1.00) | 0.33 | 34.984 | 0.517 | 1.76×10^-5^ | 0.636 |
|  | EAC | Weighted median | 0.99(0.99-1.00) | 0.86 |  |  |  |  |

**Supplementary Table S3.** Results of univariate Mendelian randomization analysis

**Abbreviation:** GERD, gastroesophageal reflux disease; BE, Barrett's esophagus; EAC, esophageal adenocarcinoma.

| **Exposure** | **Outcome** | **OR** | **P** |
| --- | --- | --- | --- |
| Cholelithiasis | GERD | 1.05(1.01-1.1) | 0.028 |
|  | BE | 1.18(1.07-1.31) | 0.0017 |
|  | EAC | 1.00(0.99-1.00) | 0.67 |
| Cholecystectomy | GERD | 1.07(1.02-1.12) | 0.0021 |
|  | BE | 1.18(1.05-1.33) | 0.0045 |
|  | EAC | 0.99(0.99-1.00) | 0.7 |

**Supplementary Table S4:** Results of multivariable Mendelian randomization analysis adjusting BMI, smoking, and alcohol assumption

**Abbreviation:** BMI, body mass index; GERD, gastroesophageal reflux disease; BE, Barrett's esophagus; EAC, esophageal adenocarcinoma.
